# Automated Spike Detection in Diverse European Wheat Plants Using Textural Features and the Frangi Filter in 2D Greenhouse Images

**DOI:** 10.3389/fpls.2020.00666

**Published:** 2020-06-23

**Authors:** Narendra Narisetti, Kerstin Neumann, Marion S. Röder, Evgeny Gladilin

**Affiliations:** ^1^Molecular Genetics, Leibniz Institute of Plant Genetics and Crop Plant Research (IPK), Gatersleben, Germany; ^2^Department of Genebank, Leibniz Institute of Plant Genetics and Crop Plant Research (IPK), Gatersleben, Germany

**Keywords:** plant phenotyping, high-throughput analysis, cultivars, spike detection, heading time point (HTP), texture, image segmentation, spike area

## Abstract

Spike is one of the crop yield organs in wheat plants. Determination of the phenological stages, including heading time point (HTP), and area of spike from non-invasive phenotyping images provides the necessary information for the inference of growth-related traits. The algorithm previously developed by Qiongyan et al. for spike detection in 2-D images turns out to be less accurate when applied to the European cultivars that produce many more leaves. Therefore, we here present an improved and extended method where (i) wavelet amplitude is used as an input to the Laws texture energy-based neural network instead of original grayscale images and (ii) non-spike structures (e.g., leaves) are subsequently suppressed by combining the result of the neural network prediction with a Frangi-filtered image. Using this two-step approach, a 98.6% overall accuracy of neural network segmentation based on direct comparison with ground-truth data could be achieved. Moreover, the comparative error rate in spike HTP detection and growth correlation among the ground truth, the algorithm developed by Qiongyan et al., and the proposed algorithm are discussed in this paper. The proposed algorithm was also capable of significantly reducing the error rate of the HTP detection by 75% and improving the accuracy of spike area estimation by 50% in comparison with the Qionagyan et al. method. With these algorithmic improvements, HTP detection on a diverse set of 369 plants was performed in a high-throughput manner. This analysis demonstrated that the HTP of 104 plants (comprises of 57 genotypes) with lower biomass and tillering range (e.g., earlier-heading types) were correctly determined. However, fine-tuning or extension of the developed method is required for high biomass plants where spike emerges within green bushes. In conclusion, our proposed method allows significantly more reliable results for HTP detection and spike growth analysis to be achieved in application to European cultivars with earlier-heading types.

## 1. Introduction

Wheat is one of the major crop species in the world, with 762 million tons of grain produced annually (FAOSTAT 2018) and providing ≥ 20% of the world's calorie and protein demand (Braun et al., 2010). However, the increasing world population and climate change are major threats to sustainable crop production (Tester and Langridge, 2010). Therefore, concentrated efforts are required to increase crop yield and production to meet future needs. Information-based plant breeding and precision agriculture are fundamental for identifying suitable wheat varieties to increase wheat productivity and production. One of the important components in both crop breeding and precision agriculture is the monitoring of plant developmental growth stages to apply informed-decision-based treatments in field or greenhouse experiments. Phenology influences grain yield components both directly and indirectly (Snape et al., 2001; Zhang et al., 2009), and in this aspect, quantitative assessment of crop phenology plays an important role in precision phenotyping as a quantifier of crop performance.

According to the Feekes scale, wheat growth can be classified into four major growth stages: tillering, stem elongation, heading, and ripening. A more detailed sub-classification is made in the BBCH scale (Witzenberger and Hack, 1989), with BBCH classes 49–59 representing phenology from heading to flowering. The determination of phenological stages is necessary for the interpretation of growth-related traits and stress tolerance acquired from non-invasive phenotyping. It is well-known that the major flowering time gene *PPD-H1* has a direct influence on leaf growth in barley (Digel et al., 2016), and flowering time genes have an impact on abiotic stress tolerance (Habte et al., 2014; Abdel-Ghani et al., 2019). In a study employing non-invasive phenotyping of barley growth, correlation of biomass and tipping time (BBCH49) was high (Neumann et al., 2017) and resulted in a constitutive biomass QTL in the region of *PPD-H1* (Dhanagond et al., 2019). However, tipping time had to be assessed by a time-consuming visual inspection of individual plant images across time. The relationship of biomass to flowering time also holds true for wheat: both crops have delayed flowering in an environment with long growing seasons to allow longer and higher vegetative growth (Cockram et al., 2007). Similar to barley, sensitive or insensitive Ppd-D1 alleles in wheat have been shown to correspond to differences in leaf area (Guo et al., 2018). In winter wheat, an earlier flowering time of semidwarf cultivars was associated with reduced biomass at anthesis (Maeoka et al., 2020). In dryland regions, simulations showed that higher yield derives from an increased biomass before anthesis leading to an increased grain number (Zhao et al., 2019). Non-invasive imaging experiments with a large wheat collection have been conducted to genetically dissect drought and heat-stress tolerance (unpublished data). An automated solution is urgently required for an effective determination of flowering time-related growth stages through non-invasive imaging.

As a first step, a reliable method for spike detection is needed. Once this is established, the time point of the first detection of spikes across a time course can be determined. To date, there have been relatively few studies concerned with wheat spike detection and growth analysis from digital images. Most of them are based on single spikes and needed to cut off spikes to classify different wheat varieties using morphological image processing algorithms, Hu moments, and neural networks (Kun et al., 2011; Bi et al., 2010, 2011). However, these methods are unsuitable for non-invasively detecting spikes from a whole plant with overlapping of leaves and young developing spikes in a high-throughput manner.

**Figure 1:**
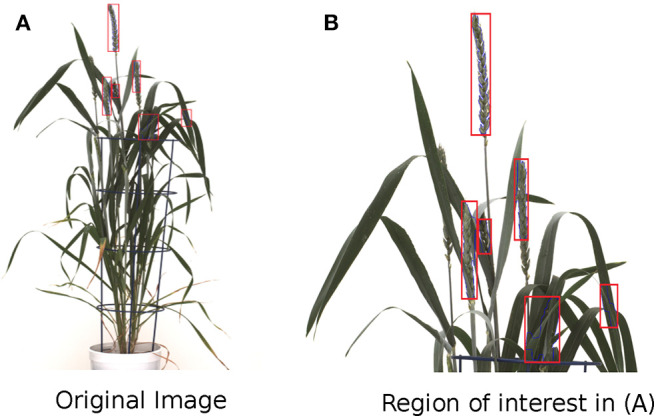
Limitations of wheat spike detection using the Qiongyan et al. (2017) algorithm. **(A)** The detection of spike and non-spike pixels in the wheat plant. **(B)** Zoomed version of detected pixels in **(A)**.

Qiongyan et al. (2017) proposed a novel approach for detecting (young) spikes in digital images of wheat plants based on Law's textural (energy) features and a neural network. This approach is based on the fact that spikes and leaves have a high color similarity but differ clearly in texture. However, when we applied this algorithm to one of our data sets, it turned out to be sensitive to the high-energy leaf edges and tillers, which led to false classifications of spike and non-spike pixels (or noisy pixels) as shown in Figure 1. However, their method was based on four Australian wheat varieties. In contrast, our data set is based on a diverse collection of high-yielding mainly European elite cultivars that are much more diverse in their plant architecture and produce more leaves and biomass compared to Australian genotypes. Accordingly, due to the presence of noisy pixels in the final image segmentation, the heading time point (HTP) BBCH55 was detected too early on our dataset compared to the ground truth data using their method. Thus, solely depending on Law's textural features lead to false detection of spikes in our wheat panel. Therefore, to overcome these artifacts, an improved and extended novel approach is proposed in this paper.

The paper is structured as follows. Section 2 deals with the improved methodological framework of spike detection, including data preparation, segmentation, and post-processing algorithms. Section 3 describes the improvement of our algorithm compared to the existing method for HTP detection and the spike growth analysis. In summary (section 4), we draw conclusions regarding the performance of our algorithm and discuss its future improvements.

## 2. Materials and Methods

### 2.1. Dataset

We used images from one experiment with 260 diverse winter wheat cultivars of mainly Central European origin. Of these lines, 220 correspond to the collection described in Voss-Fels et al. (2019) and represent high-yielding cultivars of the past decades. The remaining 40 lines are winter wheat elite cultivars from the Gabi-Wheat collection (Zanke et al., 2014), representing a similar breeding pool. Each cultivar was represented by two biological replicates. Sowing was done in small turf trays, and 14 days after sowing (DAS) at about the 2-leaf stage, plants were placed for vernalization into a growth chamber with an 8-h light period and 4°C day/night. After 8 weeks of vernalization, turf trays were placed in a greenhouse with 15-h light and 16°/12°C during the day/night for 3 days to acclimate the plants to higher temperatures. The plants were then repotted from the trays to 2-l-volume pots and were grown in the same greenhouse for another 7 days before they were placed on the imaging system, a LemnaTec 3D Scanalyzer (LemnaTec GmbH, Aachen, Germany). They were imaged and watered daily, with watering by target weight option corresponding to 89% of the plant-available water content in the soil (Dhanagond et al., 2019). Temperatures in the greenhouse of the imaging system were raised over the time course of the experiment from 16°/12°C in four steps to 30°/20°C to simulate a German spring/summer growing period, including 10 days of heat stress. In total, plants lasted 50 days on the imaging system before they were transferred to a normal greenhouse at 130 days after sowing (DAS) to grow to maturity and to evaluate the yield components. During the imaging period, the tiller number per plant was counted manually at the end of the heat period (at 125 DAS).

Images were taken from three side view angles (0°, 45°, and 90°) and one top view using RGB cameras. The top view camera (a Manta G-504) had a resolution of 2,452 × 2,056 pixels with a pixel size of 3.45 × 3.45 μ*m*, while the side view camera had a resolution of 6,576 × 4,384 pixels and a pixel size of 5.5 × 5.5 μ*m*. Plant images were later visually inspected to determine the time point of heading when the ear was half out of the flag leaf (BBCH55). Here, top view images turned out not to be suitable as, from the top, an emerging ear has a very low visible area and might be easily hidden under a bending leaf. Moreover, it is hard to define how much of the ear is above the flag leaves. Therefore, this determination was done on inspecting the three side view images. In this case, only the pots were rotated; the camera is stable. Out of all 520 plants, 369 reached BBCH55 during the imaging period belonging to 202 different cultivars. These 369 plants from 202 genotypes were available for testing our spike detection algorithm. These plants exhibit strong differences in plant architecture and are challenging for this kind of analysis, for example, spikes with or without awns, short and tall plants (plant height range at harvest time from 34 to 119 cm), and especially low and high tillering genotypes ranging from 1 to 38 tillers per plant counted at 125 DAS during the imaging period. Further, the data set exhibits differences in BBCH55 timing of 29 days.

### 2.2. Methodology

**Figure 2:**
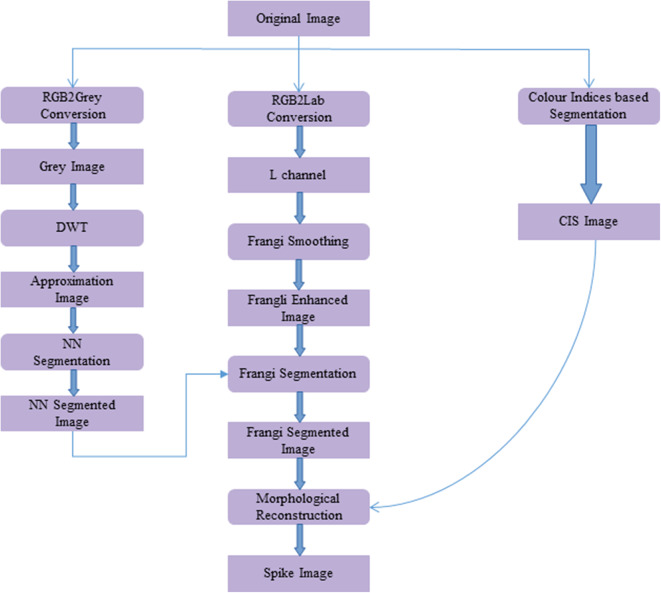
Workflow of the proposed spike detection algorithm using image processing methods. Framed rectangles represent the data modalities, and other rectangles describe the image processing operations.

**Figure 3:**
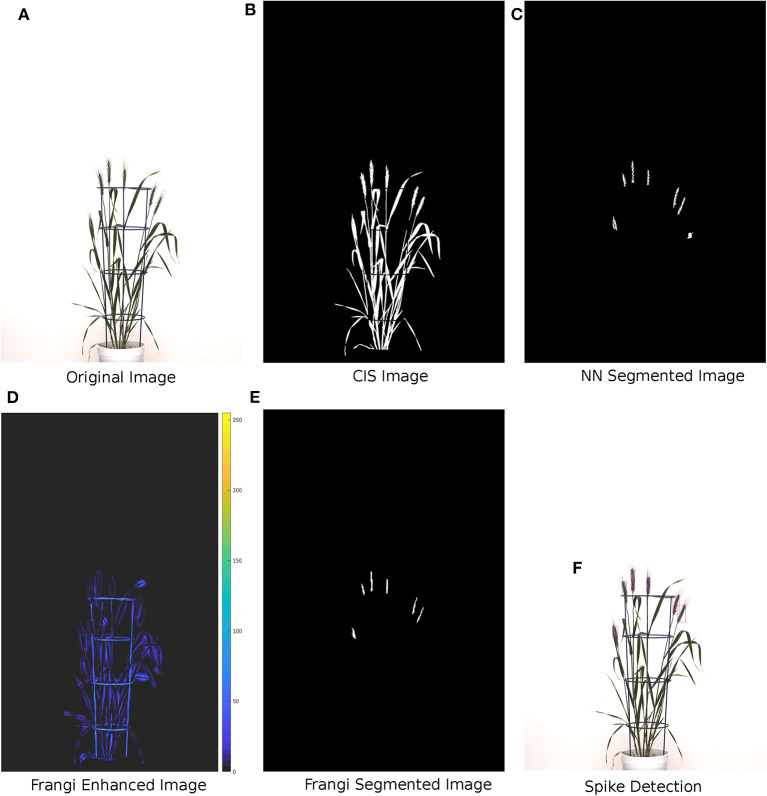
Methodology of the proposed spike detection algorithm with example images. **(A)** Wheat plant with ID 1817KN373 at 150 days after sowing. **(B)** Green color indices-based segmented image. **(C)** DWT + Laws textural features-based NN segmented image. **(D)** Image Frangi enhanced at multiple scales and orientations. **(E)** Final binary segmentation: one leaf-crossing artifact is suppressed with the Frangi-enhanced image. **(F)** Spikes detected after morphological reconstruction.

The workflow for spike detection following image acquisition is shown in Figure 2. This algorithm was developed in the MATLAB environment (MATLAB 2019a). The methodology involved in the proposed algorithm is as follows:

In the initial step, the original image (Figure 3A) is converted to a grayscale image using MATLAB's *rgb2gray* routine. To enhance the separability between the plant and background pixels, discrete wavelet transform (DWT) is applied in the preprocessing step using the Haar basis function (Stanković and Falkowski, 2003). The DWT is a single level 2-D wavelet decomposition that produces a featured image called an approximation coefficients image (A). This image is half the size of the original image and is useful for characterizing unique textures. Then, a neural network-based Laws texture energy method is applied to image A, as proposed in Bi et al. (2010) and Qiongyan et al. (2017), to segment the spike pixels from the plant pixels. Here, the segmentation of plant pixels from the background is called color index-based segmentation (CIS). Example images of the CIS and the neural network segmentation are shown in Figures 3B,C, respectively. However, the Laws texture energy is sensitive to the high-energy noisy edges (or pixels on leaves and leaf crossings) in the plant. To eliminate those noisy edges, a combination of a multi-scale Frangi-filtered image (Frangi et al., 1998) and the neural network segmented image is considered. Because the Frangi filter delivers a strength estimate of edges in the image, noisy edges can be suppressed by smoothing the image over multiple scales and orientations (Frangi et al., 1998). Therefore, this combination suppresses the tiny leaf edges and leaf crossings in the segmented image. Here, the Frangi filter is applied to an L component of the L*a*b color space image because the intensity values in the L component are closely matched with the human perception and contrast between the plant and non-plant pixels is high compared to in the a and b channels.

The Frangi-filtered image is considered one of the post-processing steps, because as a pre-processing step, it might lead to false representation of textures in the image. In other words, there might be a possibility of suppressing the spike pixels, hence modifying the unique textural characteristics of the spikes and leaves. Examples of a Frangi-filtered image and a segmented image are shown in Figures 3D,E, respectively. The complete spike is then recovered by applying morphological binary operations to the Frangi segmented image, as shown in Figure 3F.

#### 2.2.1. Wavelet Decomposition

**Figure 4:**
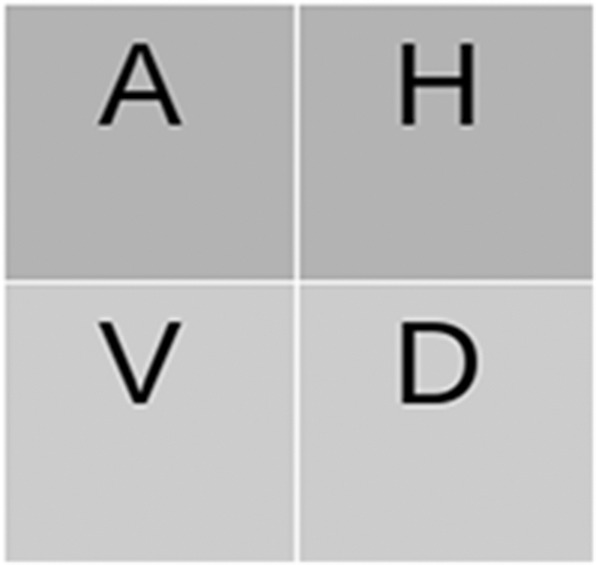
DWT Decomposition: The coefficient image (A) is again decomposed in multilevel DWT decomposition.

The wavelet-based texture classification is important because (1) it decorrelates the data (Fan, 2003) by stretching the color differences between plant and non-plant pixels in the image, and (2) it provides a non-redundant compressed image, which reduces the computation complexity significantly compared to the original grayscale image. Typically, wavelets are defined for 1-D signals, so extension to 2-D signals is usually performed by using a product of 1-D filters. The practical implementation of the wavelet transforms using different filters is as follows.

(1) $$\begin{array}{l} \begin{array}{c} A={\left[L_{x}*{\left[L_{y}*I\right]}_{↓2,1}\right]}_{↓1,2}\\ H={\left[L_{x}*{\left[G_{y}*I\right]}_{↓2,1}\right]}_{↓1,2}\\ V={\left[G_{x}*{\left[L_{y}*I\right]}_{↓2,1}\right]}_{↓1,2}\\ D={\left[G_{x}*{\left[G_{y}*I\right]}_{↓2,1}\right]}_{↓1,2} \end{array} \end{array}$$

where * denotes the convolution operator, and (↓2, 1) and (↓1, 2) represent the downscaling along rows and columns, respectively. L and G are the low- and high-pass filters, and I is the original image. The DWT decomposes an image into four sub-bands called approximation coefficients (A), horizontal (H), vertical (V), and diagonal (D), as shown in Figure 4. Sub-band A is obtained by the low-pass filtering and is accordingly called the low-resolution image, the size of which is dependent on the level of decomposition and input image size. In contrast, H, V, and D are obtained by bandpass filtering in a specific direction. Therefore, they provide detailed directional information for the image. Among these sub-bands, A is an essential feature image (or coefficients image) bearing the textural information relevant to image segmentation. Consequently, the A wavelet coefficient image is used here for texture characterization.

#### 2.2.2. Laws Textural Features-Based Image Segmentation Using Neural Networks

Laws' texture energy method (Laws, 1980) is a classical pixel-wise textural analysis approach and it has been used in many applications (Chang and Kuo, 1993; Jiang and Chen, 1998; Christodoulou et al., 2003; Mougiakakou et al., 2007). This approach uses 1-D local masks to detect various types of micro-structural textural features. The typical 1-D local masks are level detection, edge detection, and spot detection, as shown in Equation (2). However, the image is two-dimensional and requires 2-D masks for textural analysis.

(2) $$\begin{array}{l} \begin{array}{c} L3=[1\;2\;1]\text{ - }Level\;detection\\ \;E3=[\text{-}1\;0\;1]\text{ - }Edge\;Detection\\ \;S3=[\text{-}1\;2\text{ -}1]\text{ - }Spot\;Detection \end{array} \end{array}$$

The 2-D masks are generated from the 1-D masks by convolving the vertical 1-D mask with the horizontal 1-D mask. For example, mask S3L3 is calculated by convolving vertical mask S3 with horizontal mask L3 and is a zero-sum mask. In contrast, mask L3L3 is a non-zero-sum mask, which is not considered for the textural analysis. The list of 2-D masks used for the textural analysis is as follows:

(3) $$\begin{array}{l} \begin{array}{cc} L3E3=L3^{T}*E3; & E3S3=E3^{T}*E3;\\ L3S3=L3^{T}*S3; & S3L3=S3^{T}*L3;\\ E3L3=E3^{T}*L3; & S3E3=S3^{T}*E3;\\ E3E3=E3^{T}*E3; & S3S3=S3^{T}*S3; \end{array} \end{array}$$

The textural features are calculated in two steps (Chang and Kuo, 1993) using 2-D masks. In the first step, the input image is convolved with all of the above 2-D masks. Then, each individual resulting image is normalized with a unit standard deviation and average mean over the window size of 25. Consequently, eight textural feature images are generated for every input image. However, these feature images have both plant and background pixels, which increases the computational complexity of the neural network for spike detection.

To overcome the computational complexity of the image segmentation, the plant pixels (PP) are segmented from the background pixels using the CIS method (Bi et al., 2010) as follows.

(4) $$\begin{array}{l} PP=2g-r-b \end{array}$$

This method decorrelates the dominating green plant pixels from the background. A binary plant image is then generated using the binarization technique (pixel value > 0), see Figure 3B. As a result, the number of pixels for the neural network-based segmentation is reduced significantly.

The neural network is used to perform the classification of spike and non-spike pixels in the study. In practice, the neural network is trained with a large quantity of spike and non-spike pixels from the different wheat plants. The trained neural network parameters are then adapted to perform the spike detection in an automated manner. Here, a total of 218282 spike and 731054 non-spike pixels were extracted from 150 manually segmented images and subsequently used for training, testing, and validation of a network model in the sample proportion 70:15:15. The performance of the network model, with eight input nodes, one hidden layer with 10 hidden nodes, and 2 output nodes, was assessed using the conventional confusion matrix [TP FP; FN TN], components of which indicate the total number of correctly and incorrectly classified spike and non-spike pixels, respectively. The true positive (TP) and true negative (TN) rates, as well as the overall accuracy (TP+TN)/(TP+FP+FN+TN), are summarized in Table 1.

**Table 1:** Statistical performance of the neural network in the training stage.

	**Training**	**Testing**	**Validation**	**Total**
Spike pixels	152793	32773	32716	218282
Non-spike pixels	511743	109627	109684	731054
TP rate (%)	96.2	96.4	96.0	96.2
TN rate (%)	99.3	99.3	99.3	99.3
Accuracy (%)	98.5	98.6	98.5	98.6

#### 2.2.3. Frangi Filter Enhancement

The Frangi filter is a multi-scale second-order vessel enhancement method developed by Frangi et al. (1998) that is frequently used in biomedical applications (Vazquez et al., 2001; Budai et al., 2013; Shahid and Taj, 2018). The Frangi filter is used for enhancement of high-contrast vessel structures or edges along with the suppression of the non-vessel structures and thin vessel edges. Since wheat shoots have multiple leaf crossings, they exhibit vessel-like thin structures producing high-energy signals similar to spikes. In turn, this can lead to false spike detection at leaf crossings by the network model, as shown in Figure 1. The Frangi filter is applied to suppress edges resulting from such leaf crossings in the neural network segmented images.

Frangi-based vessel enhancement is achieved based on Hessian and eigenvalues. The Hessian matrix of image I is computed as follows:

(5) $$\begin{array}{l} H=\left[\begin{array}{cc} h_{11} & h_{12}\\ h_{21} & h_{22} \end{array}\right]=\sigma \;\left[\begin{array}{cc} \frac{\partial^{2}I}{\partial_{x^{2}}} & \frac{\partial^{2}I}{\partial_{x}\partial_{y}}\\ \frac{\partial^{2}I}{\partial_{y}\partial_{x}} & \frac{\partial^{2}I}{\partial_{y^{2}}} \end{array}\right] \end{array}$$

where *h*_11_, *h*_12_, *h*_21_, *h*_22_ are the second-order partial derivatives of the image and σ denotes a variable scaling factor.

To extract information on structural patterns from the Hessian matrix, the eigenvalues λ_1_ and λ_2_ are calculated, while σ is used for the enhancement of structures at different scales, see Table 2. Since we are interested in detecting and suppressing the bright vessel-like structures in the plant leaves, the image enhancement is performed under the assumption that a pixel belonging to a vessel region should have a very low value of λ_1_ and a very high magnitude of λ_2_; see Equation (6). Furthermore, the bright vessel-like structures emerge with negative λ_2_, and the filter response of the corresponding pixel with λ_2_ > 0 is considered to be zero in the enhanced image.

**Table 2:** Possible structural patterns in 2D images depending on eigenvalues λ_1_ and λ_2_.

**λ_1_**	**λ_2_**	**Structure pattern**
N	N	Noisy, no preferred direction
L	H−	Vessel structure (bright)
L	H+	Vessel structure (dark)
H−	H−	Blob like structure (bright)
H+	H+	Blob like structure (dark)

*H = high, L = low, +/− indicates the sign of the eigenvalue (Frangi et al., 1998)*.

(6) $$\begin{array}{l} |\lambda_{1}|\leq |\lambda_{2}| \end{array}$$

The enhanced image is defined as follows:

(7) $$\begin{array}{l} I_{E}=\left\{ \begin{array}{cc} \text{if}\lambda_{2}>0: & 0,\\ \text{otherwise}: & exp\left(\frac{-R_{B}^{2}}{2\beta^{2}}\right)\left(1-exp\left(\frac{-S^{2}}{2c^{2}}\right)\right) \end{array}\right. \end{array}$$

where $R_{B}=\frac{\lambda_{1}}{\lambda_{2}}$, $S=\sqrt{\lambda_{1}^{2}+\lambda_{2}^{2}}$, and c, β are constants that control the sensitivity of the filter. The enhanced image *I*_*E*_ is obtained at various scales, i.e., σ = 1, 3, 5, 7, 9. Since the maximum scale approximately matches the size of the vessel to detect, the final enhanced image *I*_*FE*_ can be obtained according to Frangi et al. (1998) by taking a maximum among all scales as defined in Equation (8).

(8) $$\begin{array}{l} I_{FE}={\text{max}}_{\sigma }I_{E} \end{array}$$

**Figure 5:**
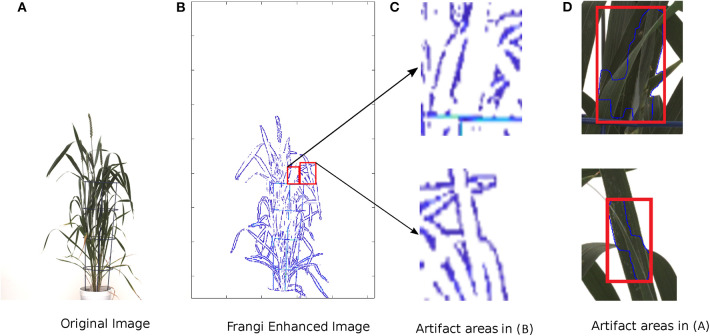
Example of suppression of leaf crossings using the Frangi Filter. From left to right: **(A)** original image of a wheat shoot, **(B)** Frangi filter-enhanced image, **(C)** examples of Frangi-enhanced regions, **(D)** examples of leaf crossings detected in the original image.

An example of edge suppression (leaf crossings) using the Frangi filter is shown in Figure 5.

Consequently, the result of the neural network segmentation is subsequently filtered under consideration of leaf-crossing regions detected by the Frangi filter (Figure 3D). This is done by eliminating the regions corresponding to leaf edges in the binary segmentation mask; see Figure 3E.

#### 2.2.4. Spike Reconstruction Using Morphological Filters

As shown in Figure 3E, only some parts of the spikes were detected using the proposed algorithm compared to the CIS image in Figure 3B. To recover the complete spikes, the logical “and” operation of the CIS image and the Frangi segmented image were performed. Then the morphological binary operations (erosion and dilation) were sequentially applied to recover the final spike area in the CIS image; see Figure 3F.

## 3. Results and Discussion

The above-described algorithm was applied to calculate the yield-related features at the transition from the tillering to flowering growth stages of wheat plants with an age of more than 90 DAS. Accordingly, the results of this study are presented in two sections dedicated to (i) detection of the time point of spike emergence and (ii) spike growth analysis from RGB images acquired using visible light cameras during an experiment with diverse winter wheat varieties. In the first section, the spike emergence was tested on 369 wheat plants from 202 different genotypes. Here, the HTP was defined as the first time in the imaging time course when the detected spike satisfied the minimum area constraint of 500 pixels. The spike area was then measured in a time series from the HTP to perform real-time growth analysis for a few selected plants.

Image analysis was performed on an Intel Xeon CPU E5-2640-based workstation running under the Linux OS. The algorithms were implemented under MATLAB 2019a (MathWorks Inc.). Training of a neural network on 949336 manually segmented spike and non-spike pixels using ten 2.40GHz CPUs with a total of 20 cores in parallel mode took approximately 80 s. The spike detection algorithm takes approximately 2.5 s to process an 8-megapixel test image. However, the processing time might vary depending on the test image size.

The root mean square error (RMSE) is used for quantification of the deviations of predictions from our model and Qiongyan et al.'s model from ground truth data,:

(9) $$\begin{array}{l} \text{RMSE}=\sqrt{\overset{n}{\underset{i=1}{\sum }}{\left(y_{i}-\hat{y_{i}}\right)}^{2}} \end{array}$$

where *y* is the ground truth value and ŷ is the model-predicted value. For consistent comparison of performance, the Qiongyan et al. model was retrained with the European cultivars.

### 3.1. Spike Emergence

The time-series images of a single plant described in Section 2 have three orientations. Accordingly, two factors are considered to estimate the HTP from multiple orientations: the spike should (1) appear in at least two orientations and (2) remain present in all days of the experiment. This means the spike or spikes should be continuously detected until the last day to avoid false emerging time points.

**Figure 6:**
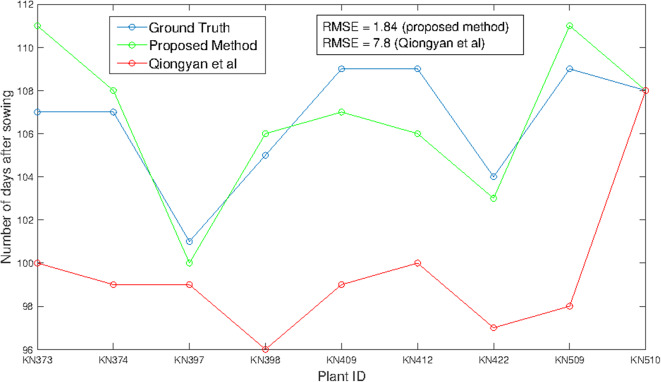
HTP detection using the method of Qiongyan et al. (2017) and our proposed method in comparison to the ground truth.

Figure 6 shows HTP detection in the wheat plant side-view images. These nine different wheat plants from the early-flowering genotypes possessing a single spike (1817KN397, 1817KN422) and multiple spikes (remaining seven plants) were considered for the training a model because we were aware that the later-flowering genotypes, which produce more biomass, will present much greater difficulties with spike visibility due to a higher probability that the spike will be covered by leaves. Figure 6 indicates that HTP values obtained by the proposed method have a significant correlation with the ground-truth HTPs, with an RMSE of 1.94, whereas the Qiongyan et al. method underperforms, with an RMSE of 7.8. This indicates that the Qiongyan et al. method is highly sensitive to the leaf artifacts whose energy is similar to that of the spike pixels but that those leaf artifacts were suppressed by the proposed method, as shown in Figure 5.

**Figure 7:**
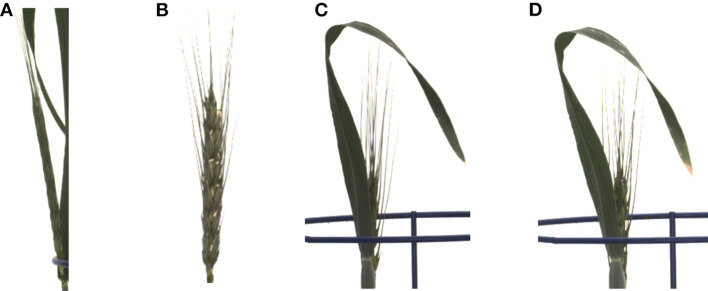
Limitations of the proposed method. **(A)** The early-stage spike texture failed to be detected in plant ID 1817KN373. **(B)** The detected spike texture in plant ID 1817KN373. **(C)** Example spike geometry less than the BBCH55 scale in plant ID 1817KN412. **(D)** Spike geometry according to the BBCH scale in plant ID 1817KN412.

On the other hand, the proposed method resulted in high HTP error rates of 4 days more and 3 days less for plant ID 1817KN373 and 1817KN412, respectively. For plant ID 1817KN373, this was because the spikes were narrow and the pixel-wise textural energy was similar to that of the leaves, as shown in Figure 7A compared to the other spikes in Figure 7B. Therefore, the HTP was detected 4 days later. In the case of plant ID 1817KN412, it turned out that the visually scored time point was determined too late, most likely by not carefully inspecting all side view angles (in the first, at 0°, the later time point looks correct, but at the 45° and 90° angles, it is visible that the earlier one is correct). Example spike images for the early HTP detection are shown in Figures 7C,D.

**Figure 8:**
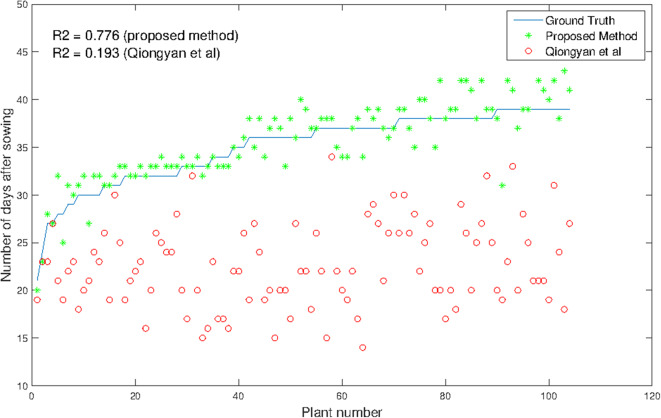
Comparison of HTP detection using our method and that of Qiongyan et al. vs. ground truth in 104 wheat plants.

The advantages and significance of the results with the proposed method showed that it is feasible for high-throughput analysis of HTP detection. Consequently, we applied the method to all 369 diverse plants in our data set that reached heading within the imaging period. As expected, 104 plants corresponding to the supposedly earlier-heading genotypes obtained a good and reliable estimation of the true heading time point. Figure 8 shows the results for the high-throughput analysis of 104 plants. It is observed that the proposed method outperforms the Qiongyan et al. method, with an *R*^2^ value of 0.776 compared to the *R*^2^ value (0.193) of the Qiongyan et al. method. Since the European elite cultivars possess more leaves, overlay artifacts result in too early HTP detection using the Qiongyan et al. method on 90% of our data. In the remaining 265 plants, the spike emerged in the final days according to the ground truth data, and they have early-stage spike textural features that are similar to the leaves. This resulted in the proposed algorithm failing to detect the spikes in the final days with a day number 0 in the output. This leads to a low value of the correlation coefficient *R*^2^ 0.0586 for the remaining 265 plants.

We compared the general plant architecture features of all 369 plants tested and classified them into three categories: (i) both plants of the genotype were classified correctly by our algorithm (94 plants from 47 genotypes, (ii) only one out the two plants of a genotype were classified correctly by our algorithm (20 plants from 10 genotypes), and (iii) none of the two plants of a genotype were classified correctly by our algorithm (Table 3; Supplementary Material). It turned out that the method performed better for earlier-flowering plants with an accordingly lower number of tillers and less biomass. Moreover, in 26 out of all 39 plants with awned spikes, heading time could be reliably estimated by our algorithm. This might arise from two factors: first, awned genotypes are more abundant in the earlier-flowering group and possess less biomass, and therefore spikes are less often hidden by leaves, and second, the model was trained based on nine early-flowering plant IDs with a bias toward awned types. Further, it might very well be that the fine awn structures, in general, help in the differentiation of the spike from the leaf background.

**Table 3:** Gene classification and comparison of architectural features of 369 plants.

**Phenotypic traits**	**Phenotypic trait mean values**		
	**2 out of 2 plants**	**1 plant of 2**	**0 out of 2 plants**
	**successful**	**successful**	**successful**
Ground truth BBCH55 (DAS)	115.5 (107–120)	118.1 (101–127)	125.5 (120–130)
Days to maturity (DAS)	175.4 (159–203)	185.2 (160–222)	193.8 (166–283)
Presence of awns (1=no, 2=yes)	1.3	1.2	1.0
Final plant height (cm)	57.1 (34–101)	64.0 (37–96)	60.9 (38–119)
Tiller number at DAS 125	7.5 (3–19)	8.4 (1–17)	11.4 (4–38)
Spike number at harvest	7.5 (3–16)	7.8 (1–14)	9.8 (4–22)
Total plant biomass at harvest	15.2 (5.7–26.8)	17.5 (4.5–28.1)	21.4 (8.1–48.0)
(grains + straw) (g)			
Total plant straw weight at harvest (g)	9.9 (3.5–15.6)	12.8 (5.7–20.0)	15.7 (5.8–38.2)

Table 3 shows mean phenotypic trait values, with minimum and maximum in brackets, of plants successfully and non-successfully classified regarding their time point of heading.

### 3.2. Spike Area

Spike area is one of the key yield measures in wheat plants, so we have examined the total spike growth of a single wheat plant in three orientations from the spike emergence day for all images. In section 3.1, nine wheat plants were considered for HTP detection. Among those, only three plants (1817KN374, 1817KN409, and 1817KN422) with a single spike and two with multiple spikes are considered for the spike growth analysis. Here, the spike area of a plant per day is calculated by taking the maximum area among the three orientations. The measured area of both algorithms is validated by the RMSE and *R*^2^. The RMSE quantifies the difference between the ground truth area and the predicted area for all days from the ear-emergence day. The *R*^2^ value compares the goodness of our proposed models and of the Qiongyan et al. model compared to the ground truth data.

**Figure 9:**
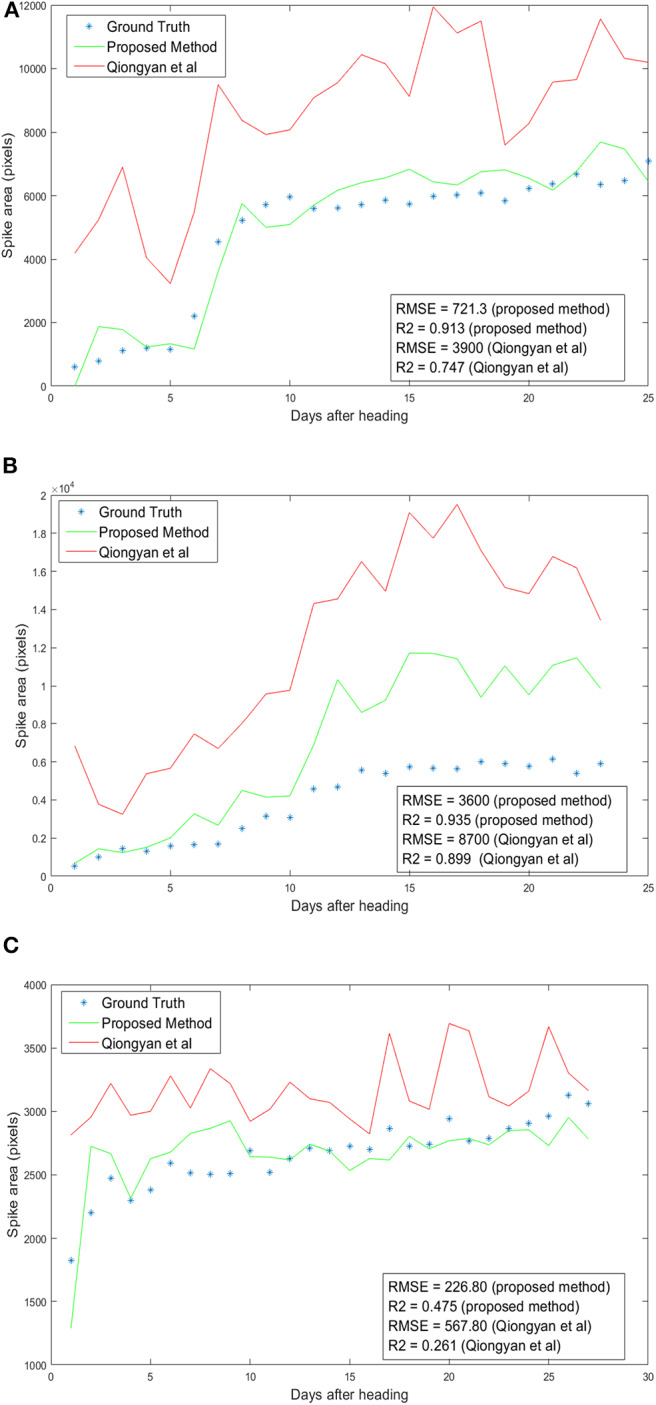
Spike growth analysis: Day number 1 represents the first day of ear emergence in the wheat plants. **(A)** Plant ID 1817KN374 with multiple spikes. **(B)** Plant ID 1817KN409 with multiple spikes. **(C)** Plant ID 1817KN422 with a single spike.

Figure 9 shows the results of spike growth analysis with the Qiongyan et al. method and with our proposed method compared to the ground truth data. Here, the ground truth data are prepared manually by segmenting the spikes using GIMP image processing software (https://www.gimp.org). The number of non-zero pixels in the segmented image represents the actual spike area or the ground-truth spike area of the image. This figure shows that the proposed method outperforms the Qiongyan et al. method overall, with a low RMSE and a high value of *R*^2^. Moreover, the RMSE is profoundly improved by more than 50% and the *R*^2^ value is significantly improved for plant ID 1817KN373 (Figure 9A) and plant ID 1817KN422 (Figure 9C). Nevertheless, plant ID 1817KN409 (Figure 9B) exhibits a high RMSE compared to the other plants in the spike growth analysis.

**Figure 10:**
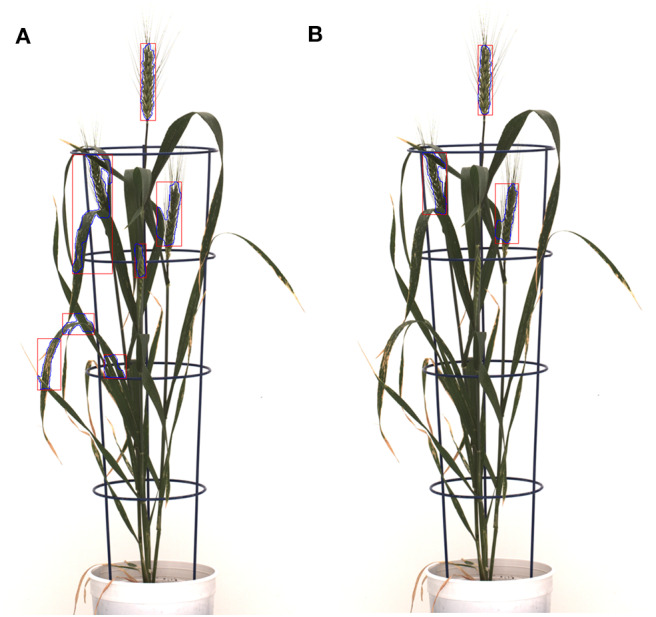
The detected leaf artifacts in **(A)** result in a high spike area compared to **(B)** for the spike growth analysis. The segmented objects are represented with blue curves and red rectangular boxes; **(A)** the Qiongyan et al. method and **(B)** the proposed method.

**Figure 11:**
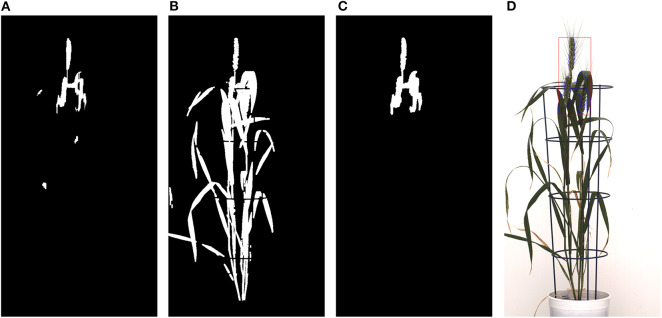
Morphological reconstruction of the spikes: **(A)** Frangi-based spike segmentation. **(B)** CIS image. **(C)** Morphologically reconstructed image using **(A,B)**. **(D)** Spikes detected in the original image represented with blue lines and a red rectangular box.

The high RMSE value for the Qiongyan et al. method is caused by the classification of leaf artifacts as spikes, which leads to an increase in the total spike area. In our method, these artifacts were eliminated using DWT and the Frangi filter. Example images of the improved spike detection are shown in Figure 10. On the other hand, the high error rate observed for plant ID 1817KN409 is due to the morphological reconstruction at the final step. This leads to the fusion of neighboring spikes with the connected stems and leaves, as shown in Figure 11.

## 4. Conclusion

Here, we present an improved method for wheat spike detection in a test data set with 369 plants from 202 diverse winter wheat varieties corresponding to mainly high-yielding Central European varieties (Voss-Fels et al., 2019). Our work relies on the algorithm proposed by Qiongyan et al. (2017), which was originally tailored to four Australian wheat varieties. By application to European elite cultivars, that earlier algorithm turned out to be too sensitive to the leaf crossing or overlay artifacts and aged leaves. This resulted in a high rate of false detection of spikes and, consequently, incorrect (too early) detection of heading time points. To overcome these limitations, we developed and evaluated an algorithmic pipeline extended by DWT and the Frangi filter that enable detection and suppression of high-energy regions caused by a high density of leaves. The proposed method has significantly improved the accuracy of the detection of spikes and the time point of heading, resulting in a reduction of the error rate (RMSE) by 75% compared to the Qiongyan et al. model. Similar improvement was also achieved in the analysis of spike growth, where the error rate of model predictions vs. ground truth data was reduced by 50% compared to Qiongyan et al. With these algorithmic improvements, detection of the heading time and analysis of spike growth can be performed in a high-throughput manner with sufficiently high accuracy.

In contrast to the majority of previous method studies, our approach was tested on a diverse set of genotypes with strong morphological differences regarding spike architecture (with or without awns), height, tiller number, biomass, and heading time. Such a data set is very challenging as it is easier to find an algorithm for identifying the plant organs in a small genotype set with much more uniform morphology. However, the biological truth is that many studies employ non-invasive phenotyping to screen genotype collections that exhibit a high phenotypic diversity (Honsdorf et al., 2014; Dhanagond et al., 2019). This requires algorithms with high performance across a highly heterogeneous background. Our proposed method represents a good starting point, as it correctly determined the heading date in 47 genotypes for both biological replicates and for at least one of the two biological replicates in a further 10 genotypes. These were mainly plants from lower biomass and tillering range and, therefore, on-average earlier heading. The method is thus expected to perform well in germplasm with relatively low biomass and tillering, as would be the case for collections from hot or dry environments. However, it also clearly showed limitations in genotypes with high biomass and high tillering (mostly later-heading types), where the spike emerges within a green “bush.” The fine-tuning or extension of the developed method for reliable spike detection in such high-biomass, high-tillering genotypes will be conducted in the near future. Further, we aim at application to other existing data sets of spring barley and spring wheat collections, where ground truth data still have to be generated. It is likely that in collections with many or exclusively awned genotypes, the method would already be applicable and yield meaningful results. It is also conceivable that the presented method will work well in bi-parental mapping populations if both parents come from the lower-biomass and tiller-number spectrum.

In conclusion, the proposed approach has the potential to predict the spike yield in other cereal plants such as barley, rice, and rye over time.

In the future, we shall explore the possibility of advancing spike detection methods in an automated manner using deep learning technologies. We also plan to perform a time series analysis of spike growth over a large experimental population (> 500 plants) to further improve the algorithm and to deliver more sophisticated solutions for plant breeders and cereal crop researchers.

## Data Availability Statement

The datasets generated for this study are available on request to the corresponding author.

## Author Contributions

NN and EG conceived, designed, and performed the computational experiments, analyzed the data, wrote the paper, prepared figures and tables, and reviewed drafts of the paper. KN and MR executed the laboratory experiments, acquired image data, wrote, and reviewed drafts of the paper.

## Conflict of Interest

The authors declare that the research was conducted in the absence of any commercial or financial relationships that could be construed as a potential conflict of interest.
